# Supplementary material for: Integration of Tmc1/2 into the mechanotransduction complex in zebrafish hair cells is regulated by Transmembrane O-methyltransferase (Tomt)
Source: eLife. 2017 May 23;6:e28474. doi: 10.7554/eLife.28474 (PMC5462536; doi:10.7554/eLife.28474)
Supplement: Supplementary file 1. — DOI: http://dx.doi.org/10.7554/eLife.28474.018 [file elife-28474-supp1.pdf]

Supplemental File 1A. Primer sequences

Sequences of primers used in this study for cloning, genotyping, mutagenesis, and RT-PCR. Where relevant, gene specific sequences are underlined.

Primer name	Purpose	Sequence
**Gateway primers**		
attB1F-comta	pDONR221-comta(NS)	GGGGACAAGTTTGTACAAAAAAGCAGGCTgcaaacATGCTGTGGGTTGTGTTGGC
attB1F-HA-GGSG-tomt_45-259	pDONR221-HA-tomt_45-259(NS)	GGGGACAAGTTTGTACAAAAAAGCAGGCTgcaaacATGTACCCATACGATGTTCCAGATTACGCT ggtggttctggtAGTCGAGAGGAACGGGCC
attB1F-mgat1a_1-110	pDONR221-mgat1a_1-110(NS)	GGGGACAAGTTTGTACAAAAAAGCAGGCTgcaaacATGCTCCGCAAGAGAAGTCCTC
attB1F-Mmu.Tomt	pDONR221-Mmu.Tomt(NS)	GGGGACAAGTTTGTACAAAAAAGCAGGCTgcaaacATGTCCCCTGCCATTGC
attB1F-tmie	pDONR221-tmie-HA	GGGGACAAGTTTGTACAAAAAAGCAGGCTgtaaacATGAGACGCGGGAGAAGAAG
attB1F-tomt	pDONR221-tomt(NS); pDONR221-tomt-HA; pDONR221-tomt_1-45(NS)	GGGGACAAGTTTGTACAAAAAAGCAGGCTAAACATGGTATCTCCTGCGATCGC
attB2F-lhfpl5a	pDONR P2R-P3-lhfpl5a	GGGGACAGCTTTCTTGTACAAAGTGGCGATGGCGAAAATGCTATCTGCC
attB2R-comta	pDONR221-comta(NS)	GGGGACCACTTTGTACAAGAAAGCTGGGTcTCCTAAGAAGACCGATTTCTCCAG
attB2R-HA+st	pDONR221-tmie-HA; pDONR221-tomt-HA	GGGGACCACTTTGTACAAGAAAGCTGGGTcttaAGCGTAATCTGGAACATCGTATGG
attB2R-mgat1a_1-110	pDONR221-mgat1a_1-110(NS)	GGGGACCACTTTGTACAAGAAAGCTGGGTcCGGAGCTGTGTGTTCAACTTCTG
attB2R-Mmu.Tomt	pDONR221-Mmu.Tomt(NS)	GGGGACCACTTTGTACAAGAAAGCTGGGTcGCCGGGTCCAGTATAGGTG
attB2R-tomt	pDONR221-tomt(NS); pDONR221-HA-tomt_45-259(NS)	GGGGACCACTTTGTACAAGAAAGCTGGGTCCTTGATGCCGATGTAAGTGAGC
attb2r-tomt_1-45	pDONR221-tomt_1-45(NS)	GGGGACCACTTTGTACAAGAAAGCTGGGTcACTGATGCCCGATAAACAGTCC
attB3R-lhfpl5a	pDONR P2R-P3-lhfpl5a	GGGGACAACTTTGTATAATAAAGTTGGTCATGCTTCCTCTTTCTTCTC
**Cloning and RT-PCR**		
lrrc51_ORF-F	RT-PCR for lrrc51	CATGTTCGGATCTTCAGTGGAC
lrrc51_ORF-R	RT-PCR for lrrc51	TCAGTCTGATTTGCTGTAACCTGTG
Mmu.Tomt_H183A-F	Site directed mutagenesis to make pDONR221-Mmu.Tomt_H183A	CCGAGGTCGGGCTGCCAGGAGCACCAGATCT
Mmu.Tomt_H183A-R	Site directed mutagenesis to make pDONR221-Mmu.Tomt_H183A	AGATCTGGTGCTCCTGGCAGCCCGACCTCGG
pcdh15a^th263b-F	genotyping pcdh15a^th263b	AGGGACTAAGCCGAAGGAAG
pcdh15a^th263b-R	genotyping pcdh15a^th263b	TCCGGCTGTAAAACATCAGC
tmc2b^sa8817-F	genotyping tmc2b^sa8817	CAAGTTATGAAATCATTTAAAGTCACTCTG
tmc2b^sa8817-R	genotyping tmc2b^sa8817	AAGAAACTTAAACAGTGAACATAATGTTGTA
tmie-F	clone tmie ORF	gtaaacATGAGACGCGGGAGAAGAAG
tmie-HA-R	clone tmie ORF C-terminally tagged with an HA epitope (lowercase)	agcgtaatctggaacatcgtatgggtaacctccaccTTTCTTCGCAGGCTTCTTGGC
tmie-R	clone tmie ORF	TCATTTCTTCGCAGGCTTCTTG
tomt_exon1-F	genotyping tomt^tk256c and tomt^nl16 alleles	TGTGTATTGCAGGTCAGTGTTG
tomt_exon1-R	genotyping tomt^tk256c and tomt^nl16 alleles	AAGCGTTTTTCTGGGTGTTG
tomt_ORF-F	RT-PCR for tomt	GTGTATTGCAGGTCAGTGTTGAAG
tomt_ORF-R	RT-PCR for tomt	GCCATATAAATGTGAGCAATGATG
tomt-HA-R	clone tomt ORF C-terminally tagged with an HA epitope (lowercase)	agcgtaatctggaacatcgtatgggtaacctccaccCTTGATGCCGATGTAAGTGAGC
NheI-kozak-Mm-Tomt	clone mouse Tomt into pcDNA3.1+	cacaGCTAGCgccaccATGTCCCCTGCCATTGC
XhoI-Mm-Tomt-HA	clone mouse Tomt-HA into pcDNA3.1+	cacaCTCGAGtcaagcgtaatctggaacatcgtatgggtaacctccaccGCCGGGTCCAGTATAGGTGAG
NheI-kozak-Mm_Comt	clone mouse Comt into pcDNA3.1+	cacaGCTAGCgccaccATGCTGTTGGCTGCTGTCT
XhoI-Mm-Comt-HA	clone mouse Comt-HA into pcDNA3.1+	cacaCTCGAGtcaagcgtaatctggaacatcgtatgggtaacctccaccGGACTTCACGGGGCTG

Supplemental File 1B. Expression constructs generated for this study

Gateway entry vectors
pDONR221-tomt(NS)
pDONR221-tomt-HA
pDONR221-tomt_1-45(NS)
pDONR221-HA-tomt_45-259(NS)
pDONR221-mgat1a_1-110(NS)
pDONR221-comta(NS)
pDONR221-Mmu.Tomt(NS)
pDONR221-Mmu.Tomt_H183A(NS)
pDONR221-tmie-HA
pDONR221-tmc2b-GFP
pDONR221-tmc1-GFP
pDONR P2R-P3-lhfpl5a

Expression constructs
pDESTCG2-myo6b:tomt-GFP
pDESTCG2-hsp70l:tomt-emGFP
pDESTCG2-myo6b:tomt-HA-pA
pDESTCG2-myo6b:Mmu.Tomt-emGFP
pDESTCG2-myo6b:Mmu.Tomt_H183A-emGFP
pDESTCG2-myo6b:comta-emGFP
pDESTCG2-myo6b:tomt_1-45-emGFP
pDESTCG2-myo6b:mgat1a_1-110-mKate2
pDESTCG2-myo6b:GFP-lhfpl5a
pDESTCG2-myo6b:tmie-HA-pA
pDESTCG2-myo6b:tmc2b-GFP-pA
pDESTCR-myo6b:tmc1-GFP-pA
pDESTCR-myo6b:tomt-p2a-nls-mCherry
pDESTCG2-myo6b:HA-tomt_45-259-emGFP
pDESTCG2-myo6b:mKate2-KDEL-pA
pcDNA3.1+Tomt-HA
pcDNA3.1+Tomt(H183A)-HA
pcDNA3.1+Comt-HA

Supplemental File 1C. Stable transgenic lines generated for this study

Transgenic Fish Lines
Tg(hsp70l:tomt-emGFP)
Tg(myo6b:comta-emGFP)
Tg(myo6b:GFP-lhfpl5a)
Tg(myo6b:mgat1a_1-110-mKate2)
Tg(myo6b:Mmu.Tomt_H183A-emGFP)
Tg(myo6b:Mmu.Tomt-emGFP)
Tg(myo6b:tmc1-GFP-pA)
Tg(myo6b:tmc2b-GFP-pA)
Tg(myo6b:tmie-HA-pA)
Tg(myo6b:tomt_1-45-emGFP)
Tg(myo6b:tomt-GFP)
Tg(myo6b:tomt-HA-pA)*

Single copy of transgene unless noted with an *
